# Diagnostics of Articular Cartilage Damage Based on Generated Acoustic Signals Using ANN—Part I: Femoral-Tibial Joint

**DOI:** 10.3390/s22062176

**Published:** 2022-03-10

**Authors:** Robert Karpiński, Przemysław Krakowski, Józef Jonak, Anna Machrowska, Marcin Maciejewski, Adam Nogalski

**Affiliations:** 1 Department of Machine Design and Mechatronics, Faculty of Mechanical Engineering, Lublin University of Technology, Nadbystrzycka 36, 20-618 Lublin, Poland; j.jonak@pollub.pl (J.J.); a.machrowska@pollub.pl (A.M.); 2 Department of Trauma Surgery and Emergency Medicine, Medical University of Lublin, Staszica 11, 20-081 Lublin, Poland; adamnogalski5@gmail.com; 3 Orthopaedic Department, Łęczna Hospital, Krasnystawska 52 str, 21-010 Łęczna, Poland; 4 Department of Electronics and Information Technology, Faculty of Electrical Engineering and Computer Science, Lublin University of Technology, Nadbystrzycka 36, 20-618 Lublin, Poland; m.maciejewski@pollub.pl

**Keywords:** vibroacoustic signal, osteoarthritis, femoral-tibial joint, kinetic chain, artificial neural network, multilevel perceptron, RBF

## Abstract

Osteoarthritis (OA) is a chronic, progressive disease which has over 300 million cases each year. Some of the main symptoms of OA are pain, restriction of joint motion and stiffness of the joint. Early diagnosis and treatment can prolong painless joint function. Vibroarthrography (VAG) is a cheap, reproducible, non-invasive and easy-to-use tool which can be implemented in the diagnostic route. The aim of this study was to establish diagnostic accuracy and to identify the most accurate signal processing method for the detection of OA in knee joints. In this study, we have enrolled a total of 67 patients, 34 in a study group and 33 in a control group. All patients in the study group were referred for surgical treatment due to intraarticular lesions, and the control group consisted of healthy individuals without knee symptoms. Cartilage status was assessed during surgery according to the International Cartilage Repair Society (ICRS) and vibroarthrography was performed one day prior to surgery in the study group. Vibroarthrography was performed in an open and closed kinematic chain for the involved knees in the study and control group. Signals were acquired by two sensors placed on the medial and lateral joint line. Using the neighbourhood component analysis (NCA) algorithm, the selection of optimal signal measures was performed. Classification using artificial neural networks was performed for three variants: I—open kinetic chain, II—closed kinetic chain, and III—open and closed kinetic chain. Vibroarthrography showed high diagnostic accuracy in determining healthy cartilage from cartilage lesions, and the number of repetitions during examination can be reduced only to closed kinematic chain.

## 1. Introduction

Knee joint is the biggest joint in the human body; moreover, it is one of the most important joints in daily living. Due to its location and function, it is often subjected to injuries and development of osteoarthritis (OA). Osteoarthritis is a progressive joint disease which leads to pain and loss of motion [1,2]. As for now, OA is incurable, and has a progressive characteristics. The reason for that is that OA affects all joint tissues; however, it mostly affects hyaline cartilage, which has a very limited healing potential [3]. In 2017, OA accounted for 303.1 million prevalent cases [4]. Those numbers indicate a gross socioeconomic impact [5]. In the clinical setting, the knee joint is the most common site of osteoarthritis and accounts for almost four fifths of the burden of OA [4]. Even though OA is considered a disease of the elderly, it also affects younger individuals. Multiple factors have been shown to produce osteoarthritic changes in knee joints, including trauma, obesity or sport activity [6,7]. Radiological symptoms of OA could be found in 8.5% of amateur sportsman and 13% of professional sportsmen between the ages of 18 and 36 [8], which is an important finding in regard to increased sports activity in the overall population. Osteoarthritic changes were also apparent in active duty soldiers younger than 20 years of age [9]. In the early stages of the disease, there were multiple methods of treatment including surgical and conservative treatment [10,11,12,13]. Nevertheless, at the end stage of the disease, total knee arthroplasty (TKA) is the gold standard of treatment. However, the survivor rate of TKA in the 10-year period of time is 81–92% [14,15]. Therefore, many researchers seek methods of prevention and early treatment of the disease [16]. Early treatment can only be achieved if the diagnosis is established without delay. The clinical diagnosis of osteoarthritis is often problematic because the phenotype of OA is variable and there is only a poor correlation between clinical and imaging findings [17,18]. Routinely, OA diagnosis is based on a conventional X-ray with the use of 0–4 Kellgren-Lawrence scale [19]. Conventional X-rays are also used for planning TKA and follow-up after surgical procedure. Conventional X-rays evaluate the signs of OA, including the forming of osteophytes and joint space narrowing, which appear after cartilage degeneration as a response from overloaded bone. Therefore, conventional X-rays lack precision in the detection of early stages of OA in knee joints [20]. Ultrasound was also proposed as a diagnostic tool in the case of early OA [21]; however, the results of this examination are comparable with standing AP radiographs [21]. Most commonly magnetic resonance imaging (MRI) is introduced into a diagnostic path for the detection of cartilage lesions. Even though MRI is the best modality available for the detection of cartilage lesions, the reported sensitivity differs grossly in the literature and ranged from 45% up to 94% [22,23]. More recent papers suggest that MRI underestimates the extent of cartilage damage, especially in the early stages [24]. Moreover, MRI is a costly examination, which requires specialized radiologists to evaluate the extent of cartilage damage and the waiting time is relatively long. Moreover, as shown by Solivetti et al. [25], up to 20% of patients referred for MRI did not have a detailed physical examination. Early detection of OA can be achieved by measuring vibrations and sounds generated by the joint during motion, under the premise that smooth, optimally lubricated cartilage surfaces move quietly relative to each other, while rough, sub-optimally lubricated surfaces move unevenly, generating more acoustic signals and vibrations, commonly referred to as crepitus [17,26,27]. These signals are generated by transient elastic waves resulting from sudden stress redistribution in the material and can be recorded from the surface of the knee [17]. Given this information it seems important to find a diagnostic modality, which could be implemented as a screening measure for patients with suspected OA. Such diagnostic tools should be reproducible, cheap, easy in evaluation and accessible. Vibroarthrography (VAG) seems to fulfill these requirements [28,29]. VAG is a measurement of vibrations or sounds generated during the movement of the joint, while classical methods of diagnostic imaging only provide information from a static position. In 1902, Blodgett [30] showed the correlation between sounds generated by knee joints on auscultation with OA progression. Over the last century, multiple researchers have engaged in the development of more accurate methods of acoustic signal analysis. Several groups of researchers achieved over 90 percent accuracy in the detection of osteoarthritic changes in knee joints with the use of acoustic signal analysis [31]. In recent decades, numerous research groups have developed this method of evaluating articular cartilage on the basis of both acoustic [32,33,34,35,36] and vibrational signals [37,38,39,40,41]. Despite the long history of the use of vibroacoustic assessment, no clear criteria have emerged for the use of this method in widespread clinical practice. Most of the work done to date has referred to imaging diagnostic methods and physical examinations, but there is little work providing detailed confirmation of the extent of damage during surgery. Therefore, the aim of this study was to compare selected indices of acoustic signals recorded in a group of patients qualified for surgery with intraoperative confirmation of the extent of cartilage damage and signals recorded in a group of healthy subjects. A procedure was carried out to select optimal signal characteristics to create a classifier based on artificial neural networks.

## 2. Materials and Methods

### 2.1. Participants

A total of 67 patients were enrolled in the study. The study group consisted of 24 males and 43 females. The mean age of the study group was 40.84 (years). All patients were qualified for surgical treatment based on medical history, physical examination and radiological findings after a detailed evaluation was performed by a specialized orthopedic surgeon. Physical examination and VAG employed in this study was performed a day prior to the scheduled surgery; therefore, the time bias between surgery and patient evaluation was reduced. The control group consisted of healthy volunteers without any previous known knee pain or injury history. In the control group (HC), the signals recorded for both knee joints were analyzed, while in the study group (OA), only the operated knee joint was considered. The mean age of the control group was 24.10 (years). Males accounted for 27% of the control group, and females accounted for 73%. A detailed description of the groups is shown in Table 1. Both the control and study groups signed written consent for participation in the study, filled out identical questionnaires and were subjected to an identical physical examination prior to VAG acquisition. Only patients with intraarticular lesions requiring surgical treatment were enrolled in the study. Patients with meniscal or ligamentous lesions, but without chondral lesions, were excluded from the study. Exclusion criteria for control group included: any previous history of trauma or joint disease, and any positive finding on physical examination prior to VAG acquisition. The study received a positive opinion from the Bioethics Committee of the Medical University of Lublin consent number KE-0254/261/2019.

### 2.2. Physical Examination

Medical history was taken from all patients and healthy volunteers. Knee joints were assessed for alignment, signs of intraarticular effusion and pain on palpation. Passive and active range of motion (ROM) was noted. Following that joint inspection, selected special tests were introduced such as McMurray, Apley and Thessaly [42,43,44] for evaluation of meniscal lesions. Varus and valgus stress was applied for an evaluation of collateral ligament continuity. Lever sign, pivot shift, Lachman and anterior drawer test were used for an examination of anterior cruciate ligament (ACL) [45,46,47,48]. Physical examination was the main determinant in excluding healthy volunteers from the control group if any test was found to be positive.

### 2.3. Surgical Treatment

**Figure 1:**
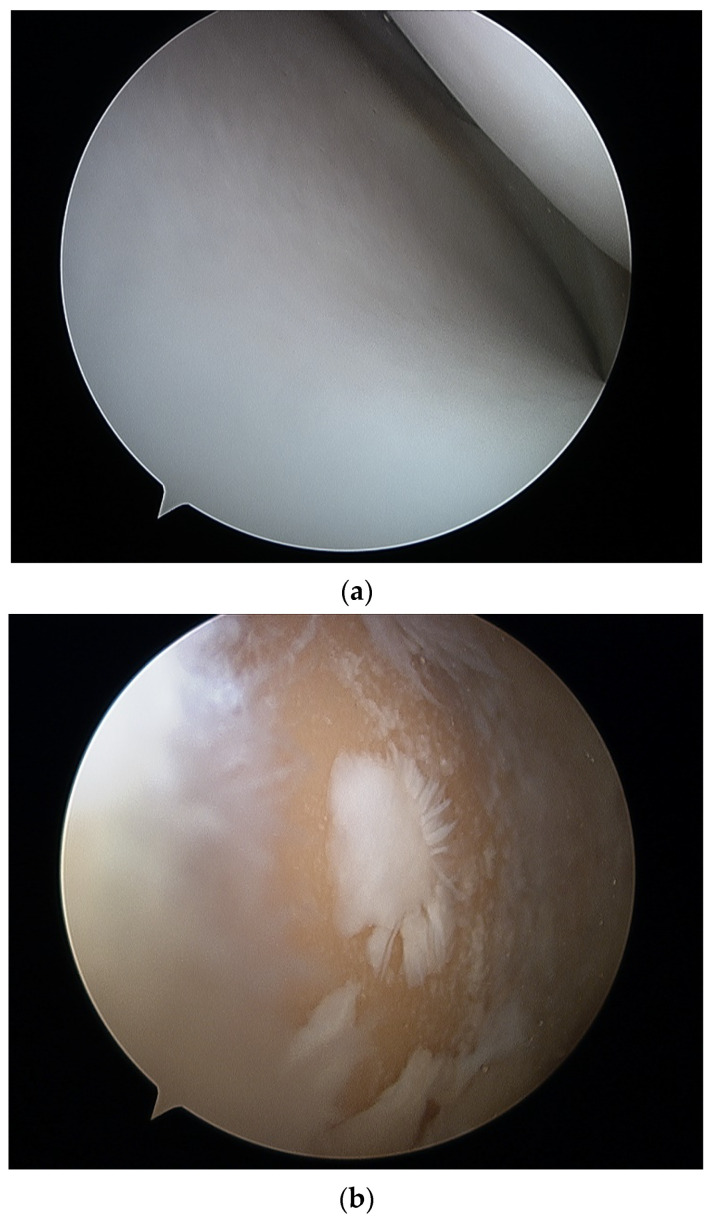
Arthroscopic view of healthy articular cartilage (**a**) and grade IV (**b**) lesion in the tibio-femoral joint.

**Figure 2:**
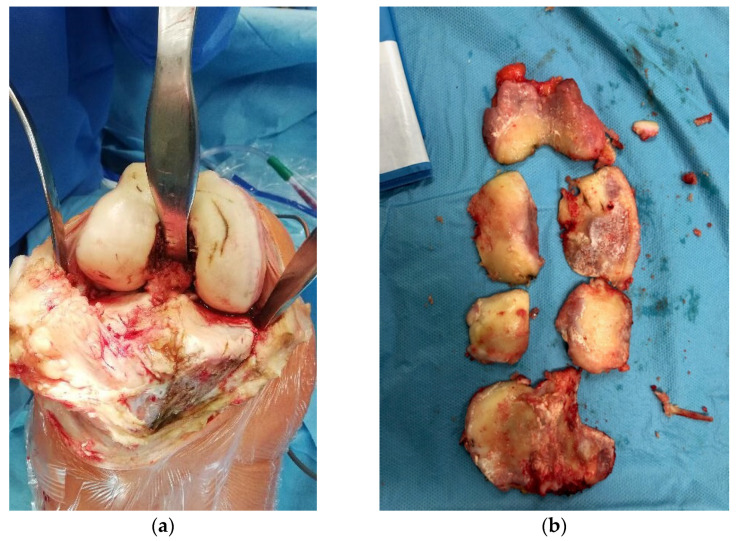
Intraoperative view prior (**a**) and after resection (**b**) of articular surfaces of the tibio-femoral joint.

All surgeries were performed by a specialized surgeon in TKA or arthroscopy according to a standard protocol depending on the type of surgery. A standard 30-degree scope was used for cartilage evaluation in arthroscopic procedures, with instruments introduced through standard anteromedial and anterolateral portals. All TKAs were performed through an anteromedial approach to the knee joint and visual evaluation of articular surfaces in cases of TKA after articular surface resection. The International Cartilage Repair Society (ICRS) grading score [49,50] was used for cartilage evaluation. Arthroscopic visualization of healthy cartilage and grade IV lesions are shown in Figure 1a,b. The intraoperative view during TKA prior and after resection of articular surfaces is shown in Figure 2a,b. 

### 2.4. Signal Acquisition 

**Figure 3:**
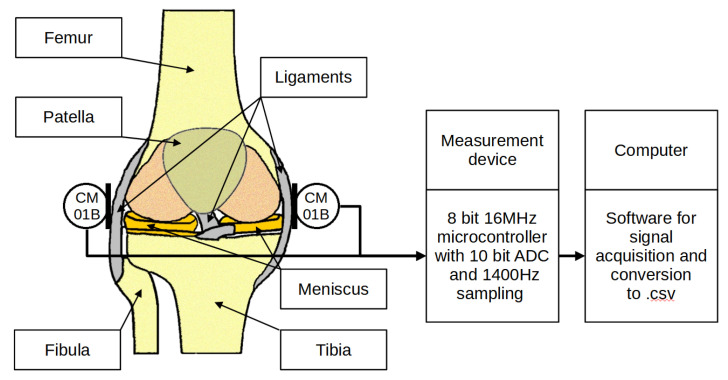
Sensor placement on the knee area and general measurement concept.

**Figure 4:**
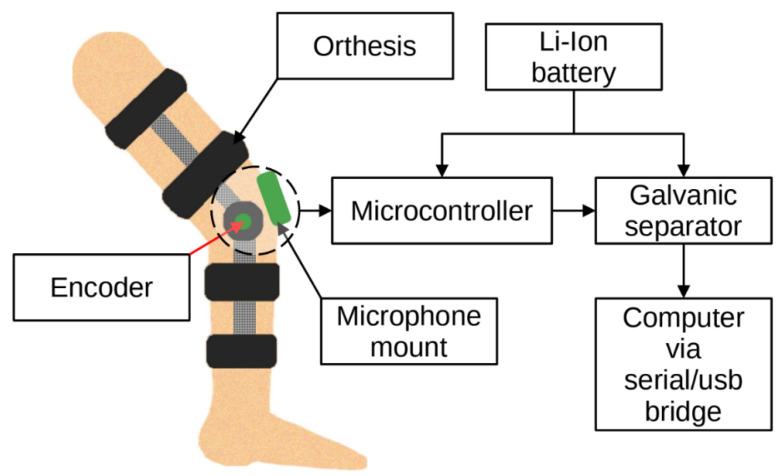
Main modules of the measurement system.

**Figure 5:**
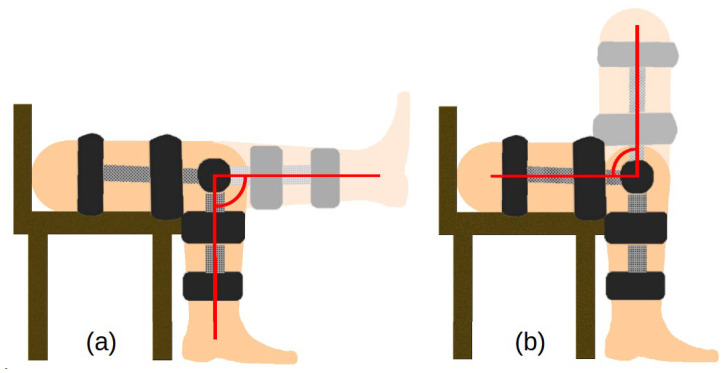
Leg movement in the open (**a**) and closed (**b**) kinematic chain.

The vibration data were acquired using a dedicated measuring system. The system consisted of three main subsystems—an orthesis, a measuring device and a computer. A dedicated orthesis with vibration transducers and a rotary encoder was placed on the patient’s knee. CM01B solid body microphones with a bandwidth from 10 Hz to 2 kHz were used [51]. The encoder used was a 10-bit Bourns [52] digital magnetic continuous encoder with 10 bit precision, giving it 1024 values per 360 degrees of rotation. The range was limited by hard stops between 0 and 90 degrees. Rotation data were sampled about ten times per second. The sensory setup was placed on a knee according to Figure 1 and connected to a dedicated measurement device consisting of several subsystems, as presented in Figure 2. First, the signal was conditioned using a preamplifier. Afterwards, the analogue signal was sampled using an 8-bit microcontroller Atmega2560 with a 10 bit ADC and a serial/usb interface. Additionally, it was necessary to provide galvanic isolation to ensure patient safety. The device uses an ADuM4160 USB 2.0 isolator chip [53] and an 11.1 V 3 s Li-Ion battery with a step-down converter and battery management system. The stream of data from the device was recorded by a computer and saved as a comma-separated value file. Measurements were taken during joint movement in an open and closed kinematic chain, as presented in Figure 3. A schematic showing the main modules of the measurement system is shown in Figure 4 while the leg motion in the open and closed kinematic chain is shown in Figure 5.

**Figure 6:**
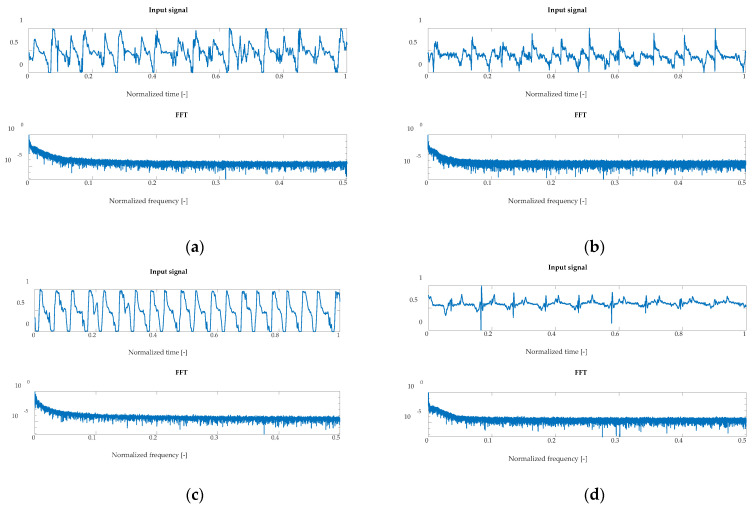
Examples of normalized signals for healthy and injured knees in the time and frequency domain for OKC and CKC recorded with a sensor placed on the lateral side. Respectively: (**a**) HC OKC, (**b**) OA OKC, (**c**) HC CKC and (**d**) OA CKC.

Examples of normalized signals for healthy and injured knees in the time and frequency domain for OKC and CKC, recorded with a sensor placed on the condyle on the lateral side is shown in Figure 6.

### 2.5. Signal Preprocessing

The collected raw VAG waveforms contain some artifacts that had to be eliminated before starting further stages of data processing, so that they did not affect the obtained results. The disturbances mainly include the registration of time series before and after the cycles of movements and random noise or interference of the electrical network. The first step in signal cleansing was to cut the time series before and after the movements that were analyzed. For this purpose, the procedure of semi-automatic signal clipping based on the data obtained from the encoder was used. Thus included automatic slope detection procedures. The introduction of manual control at the initial stage made it possible to avoid cutting off a part of the important signal for people suffering from OA with difficulties in obtaining full flexion or extension due to pain—for such people the slopes at the initial and final stages of movement are much more gentle than in the control group. 

In the case of biomedical signals, it is both important and difficult to avoid the appearance of artifacts in the form of random noise [54,55,56,57]. In order to reduce it, the ensemble empirical mode decomposition EEMD procedure was used to screen out the recorded noise and the occurring artifacts, like in [58] elaboration. The procedure for filtering signals by empirical mode decomposition EMD was introduced by Huang [59], providing a valuable tool for the analysis of nonlinear and non-stationary signals. It consists of another signal shifting, separating it into a set of Intrinsic Mode Functions (IMFs) representing signal components of high and low frequencies. The discussed procedure consists of several stages. The first is to extract the local extremes—maxima and minima—from the signal *x*(*t*). On the basis of the obtained values by means of cubic spline interpolation, two envelopes *e_up_*(*t*) and *e_dow_*(*t*) are built. Then the mean of the envelope *m*_1_(*t*) is determined:

(1) $$m_{1}\left(t\right)=\frac{e_{up}\left(t\right)+e_{dow}\left(t\right)}{2}\;.$$

Based on the above result, the signal *d*_1_(*t*) is obtained:

(2) $$d_{1}\left(t\right)=x\left(t\right)-m_{1}\left(t\right),$$

if the number of all extremes of the signal *d*_1_(*t*) and the number of zero-crossings is equal to or differs by at most one and at each point *d*_1_(*t*)*,* the mean of the constructed maxima and minima envelopes is equal to zero, it is assumed that *d*_1_(*t*) is an IMF. IMFs are frequency ordered components. After extracting *d*_1_(*t*) as IMF, the procedure is repeated for the remaining signal:

(3) $$h_{1}\left(t\right)=x\left(t\right)-d_{1}\left(t\right),$$

until the required IMF count or the stoppage criterion is reached. The monotonic signal *r*(*t*) left after the shifting process is called the residual signal. Due to the emerging mode mixing effect [60,61], manifested by leakage of frequency components between the obtained IMFs, a new approach was proposed in [62], consisting of feeding the noise-assisted signal to the input in individual samples:

(4) $$y_{n}\left(t\right)=x\left(t\right)+w_{n}\left(t\right),$$

for *n* = 1, 2, … *N*, where *N* denotes ensemble number. The signal *y_n_*(*t*) is then treated as an input signal *x*(*t*) in the classical EMD procedure. After screening, the *y_n_*(*t*) signal can be represented as:

(5) $$y_{n}\left(t\right)=\overset{i}{\underset{j=1}{\sum }}d_{j}^{n}\left(t\right)+r_{i}^{n}\left(t\right),$$

where *i* are the IMFs obtained in each decomposition, where *d_j_*(*t*) is *j*th IMF and *r_i_*(*t*) is the residual signal obtained in the *n*th decomposition. 

The data collected from sensors in real-time may have the variations or distortions due to sensor drift, which is referred to as a trend. In the conducted research, in order to reduce the quantitative and qualitative presence of artifacts, the monotonic trend *r*(*t*), extracted in the EEMD process, was removed. This preprocessed data series was subjected to further testing.

### 2.6. Feature Extraction 

In the analysis of the signal it is not necessary to focus on each momentary value change, because this is unreasonable and unattainable due to the fact that the dynamics of changes exceeds the capabilities of human perception [63,64]. It is crucial to define signal values that define the points that are most distinctive and of greatest importance to the entire waveform. 

A large number of signal features (parameters) called state indices are used in vibroacoustic diagnostics [65,66,67,68]. A summary of the parameters determined for the discrete signals used in this paper is presented below. These measures were selected based on literature data and previous studies of the authors selection of optimal signal features [28,29].

In this work, 13 feature parameters are extracted. These are:

1. Mean value (*MV*)

(6) $$\bar{x}=\frac{1}{N}\overset{N}{\underset{i=1}{\sum }}x_{i}$$

2. Straightened average value (*SA*)

The use of *SA* parameter allows to eliminate the phenomenon of approaching the mean value to zero, especially visible for oscillating signals.

(7) $$\bar{x}=\frac{1}{N}\overset{N}{\underset{i=1}{\sum }}\left|x_{i}\right|$$

3. Root mean square (*RMS*)

Root mean square is defined as the square root of the mean square. It is also known as the quadratic mean. It is a very common measure in vibroacoustic diagnostics. It is not sensitive to sudden changes manifested as single peaks in the signal. It is a parameter proportional to the vibration energy and the probability of vibration damage. *RMS* is referred to as:

(8) $$x_{RMS}=\sqrt{\frac{1}{N}\overset{N}{\underset{i=1}{\sum }}x_{i}{}^{2}}$$

4. Peak value (*PV*)

Unlike *RMS*, peak value is a parameter with a high sensitivity to rapid changes in the state of the tested objects. It is defined as:

(9) $$\hat{x}=max\left|x_{i}\right|$$

5. Peak-to-peak value (*PPV*)

*PPV* is the amplitude measured from the top-most part of the waveform to the bottom-most part of the waveform. *PPV* differs from *PV* in that two extreme values are taken into account—the smallest and the largest. It is also a measure of the dispersion of results, known in statistics as the range.

(10) $$x_{PPV}=\left|x_{max}-x_{min}\right|$$

6. Crest Factor (*CF*)

The purpose of the *CF* calculation is to give an answer of how much impacting is occurring in a waveform. Tracked over time for vibration monitoring, *CF* could be an early indicator of wear. It changes with damage development in vibroacoustic signals: as damage develops, its high value starts to decrease. In VAG studies of joint surface damage, it may be relevant by analogy to its promising results in detecting damage in mechanical bearings.

(11) $$x_{CF}=\frac{\hat{x}}{x_{RMS}}$$

7. Impact Factor (*IF*)

IF is defined in the same way as *CF*, except that the denominator is the mean value. Its diagnostic properties are also similar to those of *CF*, but it is more sensitive.

(12) $$x_{I}=\frac{\hat{x}}{\bar{x}}$$

8. Shape factor (*SF*)

In VAG analyses, *SF* could be an interesting indicator. Its changes are influenced by an increase in the deviation from the mean value.

(13) $$x_{SF}=\frac{x_{RMS}}{\bar{x}}$$

9. Variance (*VAR*)

Variance is the expected value of the squared deviation of a random variable minus its population mean. It is known as a measure of dispersion. Unlike *PPV*, it does not depend only on the two extreme values in the waveform but also contains information relating to the clustering of results around the distribution center. Values deviating from the mean value have a greater influence on this feature. For low *VAR* values, it is assumed that the results are clustered around the mean value, but for high results, they are scattered. In the context of defining the state of knee joint damage and the characteristic impulses occurring in HC group waveforms, it may be a promising indicator.

(14) $$x_{VAR}^{2}=\frac{1}{N-1}\overset{N}{\underset{i=1}{\sum }}{\left(x_{i}-\bar{x}\right)}^{2}$$

10. Kurtosis (*KUR*)

*KUR* is a parameter describing the degree of concentration of results in the distribution. It provides information about the degree of similarity of data scattered around the mean in relation to the normal distribution. The value of kurtosis for normal distribution is 3; in cases where the analyzed distribution is more pointed, this value will be higher, and for smaller values of kurtosis the distribution is flattened. According to [69], the appearance of vibration pulses in the signal causes an increase in the kurtosis value. By analogy to machine vibration diagnostics, higher values may indicate damage.

(15) $$x_{KUR}=\frac{\frac{1}{N}\sum_{i=1}^{N}{\left(x_{i}-\bar{x}\right)}^{4}}{{\left[\frac{1}{N}\sum_{i=1}^{N}{\left(x_{i}-\bar{x}\right)}^{2}\right]}^{2}}$$

11. *M6A*

The *M6A* parameter is the sixth central moment normalized by the variance raised to the third power. This factor is more sensitive to the presence of impulses in the signal. In machine diagnostics it is used for differential signals. Here it was placed experimentally for a signal subjected to preprocessing filtration.

(16) $$x_{M6A}=\frac{\frac{1}{N}\sum_{i=1}^{N}{\left(x_{i}-\bar{x}\right)}^{6}}{{\left[\frac{1}{N}\sum_{i=1}^{N}{\left(x_{i}-\bar{x}\right)}^{2}\right]}^{3}}$$

12. *M8A*

An even more sensitive parameter is *M8A,* known as the eighth central moment, normalized by the variance to the fourth power. In machine diagnostics it is used for the differential signal. Here it was placed experimentally for a signal subjected to preprocessing filtration. The feature is described as:

(17) $$x_{M8A}=\frac{\frac{1}{N}\sum_{i=1}^{N}{\left(x_{i}-\bar{x}\right)}^{8}}{{\left[\frac{1}{N}\sum_{i=1}^{N}{\left(x_{i}-\bar{x}\right)}^{2}\right]}^{4}}$$

### 2.7. Selection of Optimal Signal Features

In the process of building classification models, the use of too-large sets of measures may reduce the ability to generalize cases and unnecessarily extend the computation process. This is particularly important in the case of small sets of recorded data. In the presented paper, a neighborhood component analysis (NCA) algorithm was used to reject inappropriate and redundant features. 

NCA is one of the more interesting classification techniques. It is a non-parametric and embedded method developed based on the nearest neighbor (KNN) algorithm [70]. According to [71,72] in NCA carrying out analyses, a data set is assumed:

(18) $$S=\left\{\left(x_{i},y_{i}\right),\;i=1,2,\ldots ,N\right\},$$

where *x_i_* described *d*- dimensional vector, *yi* ∈{1,2,…,c} is its corresponding class label, *c* is the number of classes and *N* is a observations number. Then, in terms of the weight vector *w*, the weighted distance between the two samples *x_i_* and *x_j_* was determined by [71,72]:

(19) $$D_{w}\left(x_{i},x_{j}\right)=\overset{d}{\underset{l=1}{\sum }}w_{l}^{2}\left|x_{il}-x_{jl}\right|,$$

where *w_l_* is a weight associated with the *l*th feature. 

To ensure the maximum accuracy of classification, the leave-one-out technique is used on a given set of *S*. Randomly, a point is picked from *S* as a reference point and is labelled accordingly. The reference point *x* is chosen from *S* by a probability distribution, and the probability of *x_i_* selects *x_j_* as its reference point [71,72]:

(20) $$pij=\{\begin{array}{c} 0,\;\;\mathrm{if}\;i=j\\ \frac{\kappa \left(D_{w}\left(x_{i},x_{j}\right)\right)}{\sum_{k\neq i}\kappa \left(D_{w}\left(x_{i},x_{k}\right)\right)},\;\mathrm{if}\;i\neq j \end{array}\;,$$

where *κ*(*z*) *= exp*(*−z/*σ) is a kernel function and the kernel width *σ* is an input parameter that influences the probability of each of the points being selected as the reference point. The probability of the query point *x_i_* being correctly classified is given by [71,72]:

(21) $$p_{i}=\overset{N}{\underset{j-1,j\neq i}{\sum }}y_{ij}p_{ij},$$

where

(22) $$y_{ij}=\{\begin{array}{c} 1,\;\;\;\mathrm{if}\;y_{i}=y_{j}\\ 0,\;\mathrm{otherwise} \end{array}$$

Thus, the approximate accuracy of the classification omitting one output can be written as [71,72]:

(23) $$F\left(w\right)=\overset{N}{\underset{i=1}{\sum }}p_{i}$$

The aim of the NCA is to maximize *F*(*w*) with respect to w by introducing the regularized objective term *λ* [71,72]:

(24) $$F\left(w\right)=\overset{N}{\underset{i=1}{\sum }}p_{i}-\lambda \;\overset{d}{\underset{l=1}{\sum }}w_{l}{}^{2}$$

where *λ* > 0 is a regulation parameter which can be tuned via cross validation [71,72].

(25) $$F\left(w\right)=\overset{N}{\underset{i=1}{\sum }}\overset{N}{\underset{j=1,j\neq i}{\sum }}p_{ij}y_{ij}-\lambda \;\overset{d}{\underset{l=1}{\sum }}w_{l}{}^{2}$$

Since the objective function *F*(*w*) is differentiable, its derivative with respect to *w_r_* can be computed as [71,72]:

(26) $$\frac{\partial F\left(w\right)}{\partial w_{l}}=\underset{i=1}{\sum }\underset{j=1}{\sum }y_{ij}\left[\frac{2}{\sigma }p_{ij}\left(\underset{k\neq i}{\sum }p_{ik}\left|x_{il}-x_{kl}\right|-\left|x_{il}-x_{jl}\right|\right)w_{l}\right]-2\lambda w_{l}$$

(27) $$\frac{\partial F\left(w\right)}{\partial w_{l}}=\frac{2}{\sigma }\underset{i=1}{\sum }\left(p_{i}\underset{k\neq i}{\sum }p_{ik}\left|x_{il}-x_{kl}\right|-\underset{j=1}{\sum }y_{ij}p_{ij}\left|x_{il}-x_{jl}\right|\right)w_{l}-2\lambda w_{l},$$

and finally it is obtained as follows [71,72]:

(28) $$\frac{\partial F\left(w\right)}{\partial w_{l}}=2\left(\frac{1}{\sigma }\underset{i=1}{\sum }\left(p_{i}\underset{k\neq i}{\sum }p_{ij}\left|x_{il}-x_{jl}\right|-\underset{j=1}{\sum }y_{ij}p_{ij}\left|x_{il}-x_{jl}\right|\right)-\lambda \right)w_{l}$$

The above dependence allows for the update of the gradient equation. Obtaining the best *λ* corresponds to the minimum classification loss. The mean loss in the folds of the cross-validation depends on the choice of *λ*. This parameter tuning causes minimal loss of classification and has been computed by *1/N_t_*, where *N_t_* is the total number of observations in the training set. The criteria for selecting significant features using feature weights based on the definition of the relative threshold (*T*) is given by [71,72]:

(29) T=τ*max(w),

where τ is the tolerance fixed to 0.02 [71] and *w* is the updated features weight. According to [72], the features weight greater than *T* counted as significant features, and the remaining features were expelled from set *S*. When *λ* is too large, all of the feature weights approach zeros, resulting in them all constituting irrelevant features. Hence, it is essential to tune *λ* in a way that produces minimal classification loss.

### 2.8. Artificial Neural Networks 

A neural network is a collection of properly connected neurons arranged in layers. An artificial neuron from a technical point of view is an element whose properties correspond to the selected properties of its biological counterpart [73]. The input signals are multiplied by coefficients called synaptic weights. Each neuron calculates a weighted sum of its inputs, and these are then summed up. The activation level thus determined becomes the argument of the transition function (activation function), which calculates the output of the neuron [74]. The input signals are multiplied by factors called synaptic weights. The values of the weights can be changed; this allows the network to learn and adapt to the considered task. The activation level thus determined becomes the argument of the transition function (activation function), which calculates the output value of the neuron [74,75]. The most common type of networks are the MLP (Multilayer Perceptron) [76,77] and RBF (Radial Basis Function) neural networks. The number of input and output neurons depends on the complexity of the problem being solved. One of the most popular applications of neural networks is solving classification problems, in which ANN is a tool for assigning the studied objects to the correct classes [78,79].

**Figure 7:**
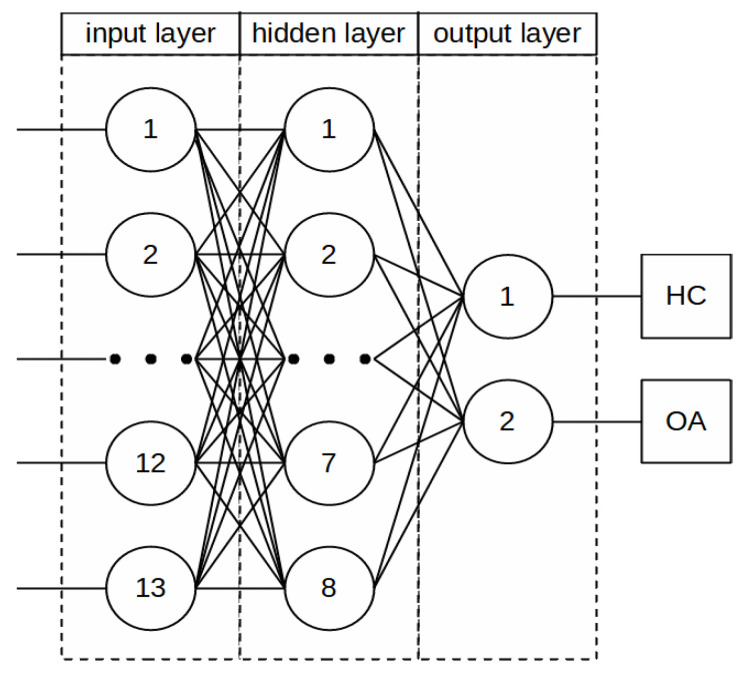
Graphical visualization of the applied neural network.

Three classification variants were considered in the study to determine the optimal testing protocols allowing the best possible diagnoses of cartilage damage. The Statistica 13.1 package (Tulsa, OK, USA) containing modules including machine learning and artificial neural networks was used for the calculations. In all variants, information such as age, gender, BMI (Body Mass Index), and selected measures of acoustic signals were provided at the inputs. Data are randomly divided into 70% training, 15% testing, and 15% validation. A 2-stage simplified classification corresponding to cartilage damage states was proposed at the outputs: 1-healthy cartilage, 2- cartilage for further diagnosis and surgical treatment (I to IV degree of damage according to the Kellgren–Lawrence scale). Variant I involved teaching the network using selected measures of signals recorded in a closed kinetic chain (CKC). Variant II involved selected measures of signals recorded in the open kinetic chain (OKC). Variant III included selected signal measures for both kinetic chains (CKC and OKC). A graphical visualization of the neural network used is shown in Figure 7.

## 3. Results and Discussion

### 3.1. Selection of Optimal Signal Features

**Figure 8:**
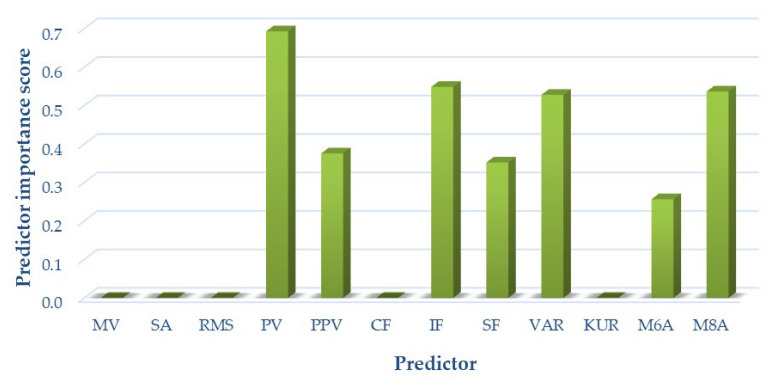
Selection of optimal features for variant I (OKC).

**Figure 9:**
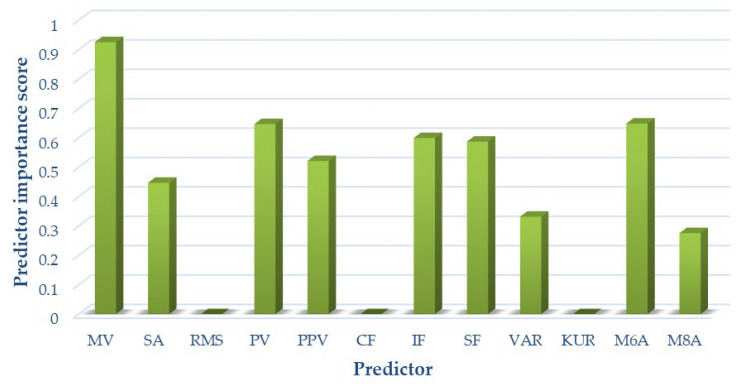
Selection of optimal features for variant II (CKC).

**Figure 10:**
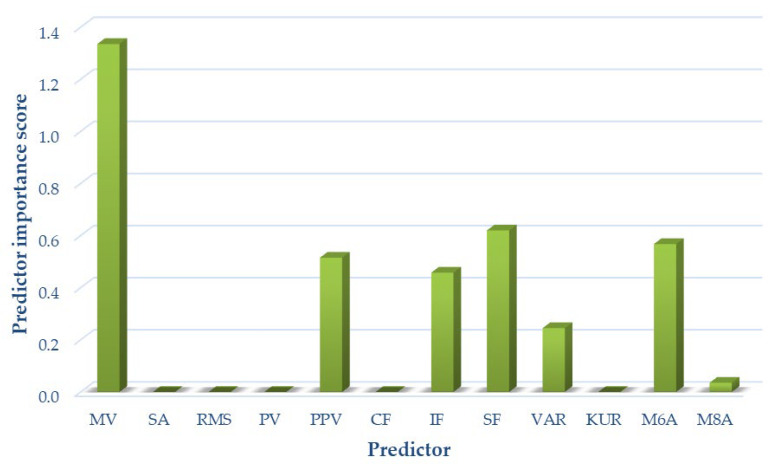
Selection of optimal features for variant III (OKC and CKC).

Due to the complex mechanics of the knee joint and significant differences occurring in articular cartilage loads, as well as the different friction and lubrication parameters in both kinetic chains, optimal signal measures were selected separately for each of the analyzed variants. The results of the analyses are shown in Figure 8 for variant I, Figure 9 for variant II, and Figure 10 for variant III, respectively. 

For variant I, the following measures of acoustic signals were selected based on the analyses performed: peak value, peak-to-peak value, impact factor, shape factor, variance, *M6A*, and *M8A*. These measures were used as input data for variant I classification. 

For variant II, the following measures of acoustic signals were selected based on the analyses performed: mean value, straightened average value, peak value, peak-to-peak value, impact factor, shape factor, variance, *M6A*, and *M8A*. These measures were used as input data in the variant II classification.

For variant III, the following measures of acoustic signals were selected based on the analyses performed: mean value, peak-to-peak value, impact factor, shape factor, variance, *M6A*, and *M8A*. These measures were used as input data for variant III classification.

### 3.2. Classification 

The results for the most accurate classifiers are presented below for one case each of multilayer perceptron (MLP) and radial basis function (RBF) networks, respectively. The models were created using the automatic network search algorithm included in the Statistica package. The quality results for each network are shown in Table 2.

In Table 2, in the network name column, we present data describing the type and structure of the network. The numerical notation following the network type describes respectively: the first value is the number of neurons in the input layer, the second is the number of neurons in the hidden layer while the third describes the number of network outputs. The next three columns report the quality of the network, separately for the learning and test data. For quality variables (classification), the relative number of correctly classified cases (relative to the total number of cases) is given. The highest quality of learning was obtained for the MLP network in variant I, in which the classification was conducted only on the basis of data obtained for a closed kinetic chain. It is a network with thirteen input neurons (thirteen variable inputs), nine neurons in the hidden layer, and two output neurons (assigning data to one of two classes of HC and OA). The learning algorithm used is BFGS 45 (Broyden–Fletcher–Goldfarb–Shanno), the error function is SOS (sum of squares), the activation function in the hidden layer is a logistic function while in the output layer it is an exponential function. The lowest quality of learning in the analyzed variants was recorded for the RBF network in variant I. In the case of testing quality, the highest value was observed for the MLP network in variant I and the RBF network in variant II, the lowest for the MLP network in variant II, respectively. The highest quality of validation was obtained for MLP and RBF networks for variant I. The information on parameters such as learning algorithm, error function, and activation functions for each network is presented in Table 2. 

The detailed results for classification in each group and overall classification accuracy for all three variants are shown in Table 3.

The best classification accuracy (97.75% of correctly assigned cases) in the HC group was obtained for the MLP network in variant I and the RBF network in variant II, the lowest accuracy in this group was obtained for the RBF network in variant III. For the OA group, the highest accuracy was obtained for the MLP network in variant I and the lowest accuracy was obtained for the RBF network in variant II. The highest classification accuracy in both groups was obtained for the MLP network for variant I, with 96.32% of correctly assigned cases, respectively. The lowest accuracy was obtained for the RBF network in variant III and it was 89.63% correctly assigned to each class of cases. 

**Figure 11:**
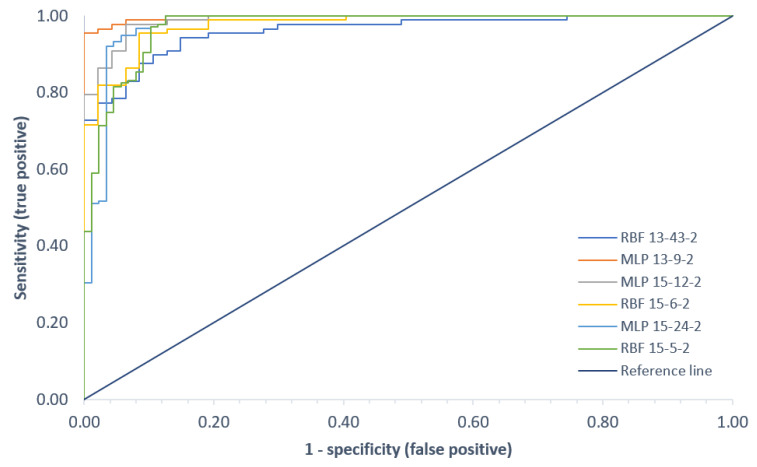
Comparison of the ROC curves for all classification variants.

In mathematical statistics, the ROC curve is a graphical representation of the effectiveness of a predictive model by plotting the qualitative characteristics of the binary classifiers produced from the model using a number of different cutoff points [80,81]. The interpretation of the Area Under the ROC (Table 4) is the probability that the predictive model under the test will rate a random positive class element higher than a random negative class element. The larger the AUROC, the better. The largest area under the ROC curve was observed for the MLP network in variant I, the smallest area for the RBF network in the same variant, respectively. A summary of the ROC curves for each classifier for all three variants is shown in Figure 11. Information on the area under the curve and the threshold values for each curve is summarized in Table 4. 

The analysis of the classification results obtained shows that there are no significant differences between variants I and II involving the analysis of movements in one kinetic chain and variant III involving the analysis for both kinetic chains together. This suggests the possibility of simplifying the procedure and limiting the test to a procedure involving the execution of repetitions in one kinetic chain, thus reducing the test time and computation time. The obtained results also show that MLP-type networks perform better in solving the studied problem. 

Our study shows that examination procedure can be reduced in the number and range of repetitions, while it shows that there is no positive correlation with the increased volume of data (two kinematic chains). Our results show that, in the case of two sensors placed medially and laterally on a joint line, there is no need for repetition in both kinematic chains. The limitation of this study is a simplification of a classification model only to two groups with healthy cartilage and cartilage lesions without distinguishing between lesion severity grades. Future studies focused on the determination of cartilage lesion grade are required and will be performed by the authors. Even though VAG is an emerging technique of cartilage evaluation, our results correspond with other author’s findings, that this diagnostic modality can be applied in an orthopaedic setting [82]. However, the best signal processing method is still to be found and popularized by engineers and the orthopaedic community. Different pathways have been proposed for testing protocols [34,83], showing good diagnostic accuracy in the field of cartilage damage. The sensitivity and specificity published by other authors range between 0.56–1 and 0.74–1 respectively [84,85,86]. Those findings correspond with our own results, which show the high sensitivity and specificity of VAG in cartilage lesion diagnostics. Nevertheless, the technique seems promising for a wide range of results and requires further studies in order to establish a reproducible protocol for orthopaedic testing. 

## 4. Conclusions

Analysis of the results shows that vibroarthrography can be an effective, low-cost and accurate diagnostic modality for the evaluation of cartilage damage in tibio-femoral joints, and can be implemented in daily orthopedic practice. A neighborhood component analysis (NCA) algorithm used for the detection of signals optimized the quantity of input data and aided in the maximizing of classification accuracy in a shorter calculation time. 

## Figures and Tables

**Table 1:** Characteristics of study participants.

Study Group	N	Males/ Females	Age (Years ± SD)	Heigh (m ± SD)	Weight (kg ± SD)	BMI	Tegner-Lyshom Score
Healthy control (HC)	33	9/24	24.10 ± 5.56	1.71 ± 0.09	65.16 ± 15.10	21.95 ± 3.09	100 ± 0.0
Osteoarthrisis (OA)	34	15/19	56.15 ± 12.99	1.69 ± 0.09	89.08 ± 14.30	31.19 ± 4.83	38.59 ± 12.96

**Table 2:** Quality of the MLP and RBF neural network for variant I (open kinetic chain), II (close kinetic chain) and III (open and close kinetic chain).

Variant	Network Name	Quality of Learning	Quality of Testing	Quality of Validation	Learning Algorithm	Error Function	Activation (Hidden)	Activation (Output)
I	MLP 13-9-2	96.32	100.00	96.43	BFGS 45	SOS	Logistic	Exponential
	RBF 13-43-2	89.71	96.43	96.43	RBFT	Entropy	Gauss	Softmax
II	MLP 15-12-2	94.85	92.86	92.86	BFGS 14	Entropy	Linear	Softmax
	RBF 15-6-2	91.91	100.00	89.29	RBFT	SOS	Gauss	Linear
III	MLP 15-24-2	93.70	94.74	85.96	BFGS 13	SOS	Linear	Linear
	RBF 15-5-2	89.63	89.47	91.23	RBFT	Entropy	Gauss	Softmax

**Table 3:** Summary of classification accuracy of MLP and RBF networks for variant I, II and III.

Network Name		HC	OA	Total
MLP 13-9-2	Total	89.00	47.00	136.00
	Correct	87.00	44.00	131.00
	Incorrect	2.00	3.00	5.00
	Correct (%)	97.75	93.62	96.32
	Incorrect (%)	2.25	6.38	3.68
RBF 13-43-2	Total	89.00	47.00	136.00
	Correct	83.00	39.00	122.00
	Incorrect	6.00	8.00	14.00
	Correct (%)	93.26	82.98	89.71
	Incorrect (%)	6.74	17.02	10.29
MLP 15-12-2	Total	89.00	47.00	136.00
	Correct	86.00	43.00	129.00
	Incorrect	3.00	4.00	7.00
	Correct (%)	96.63	91.49	94.85
	Incorrect (%)	3.37	8.51	5.15
RBF 15-6-2	Total	89.00	47.00	136.00
	Correct	87.00	38.00	125.00
	Incorrect	2.00	9.00	11.00
	Correct (%)	97.75	80.85	91.91
	Incorrect (%)	2.25	19.15	8.09
MLP 15-24-2	Total	182.00	88.00	270.00
	Correct	176.00	77.00	253.00
	Incorrect	6.00	11.00	17.00
	Correct (%)	96.70	87.50	93.70
	Incorrect (%)	3.30	12.50	6.30
RBF 15-5-2	Total	182.00	88.00	270.00
	Correct	163.00	79.00	242.00
	Incorrect	19.00	9.00	28.00
	Correct (%)	89.56	89.77	89.63
	Incorrect (%)	10.44	10.23	10.37

**Table 4:** Area under the ROC curves and ROC threshold.

	Variant I		Variant II		Variant III	
	MPL	RBF	MPL	RBF	MPL	RBF
Area Under the ROC	0.996	0.960	0.989	0.977	0.977	0.974
ROC Threshold	0.603	0.571	0.647	0.505	0.645	0.529

## Data Availability

Data presented in this study are available from corresponding authors upon request.
